# Bilateral Tubal Pregnancies Presenting 11 Days Apart: A Case Report

**DOI:** 10.5811/cpcem.2022.10.56910

**Published:** 2023-02-09

**Authors:** Leyla S. Farshidpour, David R. Vinson, Edward J. Durant

**Affiliations:** * University of California Davis School of Medicine, Sacramento, California; † The Permanente Medical Group, Division of Research, and the CREST Network, Oakland, California; ‡ Kaiser Permanente Roseville Medical Center, Department of Emergency Medicine, Roseville, California; § The Permanente Medical Group, Kaiser Permanente Bernard J. Tyson School of Medicine, and the CREST Network, Oakland, California; ¶ Kaiser Permanente Modesto Medical Center, Department of Emergency Medicine, Modesto, California

**Keywords:** case report, tubal pregnancy, ectopic pregnancy, pelvic pain, abdominal pain

## Abstract

**Introduction:**

Ectopic pregnancy is the most common cause of maternal mortality in the first trimester.^1^ Bilateral tubal pregnancy is the rarest subset with an estimated incidence of one in 725 to 1,580 ectopic pregnancies.^2^ Of the cases of bilateral tubal pregnancy reported in the literature, most were associated with the use of assisted reproductive techniques.^3^ Here we present the case of a patient, without a prior history of reproductive technology use, who underwent treatment for a tubal pregnancy and was subsequently found to have a second, contralateral tubal pregnancy 11 days later.

**Case Report:**

A 35-year-old female gravida eight para two with a history of left tubal pregnancy and salpingectomy 11 days prior, presented to the emergency department (ED) with two days of left lower and upper quadrant abdominal pain. The patient’s last menstrual period had been several months prior. A physical examination revealed left lower quadrant abdominal tenderness, rebound, guarding, and left adnexal tenderness. Her vital signs were unremarkable, and her laboratory studies revealed normal white blood cell and hemoglobin values. Her human chorionic gonadotropin had tripled from her last presentation 11 days prior. Transvaginal ultrasound showed a possible ectopic pregnancy adjacent to the right ovary. She promptly underwent a right salpingectomy. Pathology findings confirmed a tubal pregnancy, and the patient’s postoperative course was uneventful.

**Conclusion:**

This case highlights the importance of maintaining a high index of suspicion for ectopic pregnancy in all biologically female patients of reproductive age who present to the ED with abdominal pain.

## INTRODUCTION

Ectopic pregnancy, defined as any pregnancy outside the uterine cavity, is the most common cause of hemorrhage-related maternal mortality in the first trimester. Up to 90% of these pregnancies are of tubal origin. Less common sites of implantation include the ovary, cesarean scar, cervix, and abdomen.^1^ Bilateral tubal pregnancy is the rarest subset, with an estimated incidence of one out of every 725 to 1,580 ectopic pregnancies and one out of every 200,000 spontaneous ectopic pregnancies.^2^ The risk of heterotopic pregnancy, where an ectopic and intrauterine pregnancy occur together, is estimated to range from one in 4,000 to one in 30,000 in natural conception.^4^ Several identified risk factors increase the likelihood of ectopic pregnancy, including previous damage to the fallopian tubes from prior ascending pelvic infections or surgeries, multiple embryo transfers in assisted reproductive technology, history of cigarette smoking, and advanced age.^4^ Interestingly, half of all individuals diagnosed with an ectopic pregnancy have no known risk factors. The prompt identification of ectopic pregnancy is especially important as ruptured ectopic pregnancies continue to account for 2.7% of all pregnancy-related deaths and thus are true medical emergencies.^4^ Here we present a case of a patient who underwent treatment for a tubal pregnancy and was subsequently found to have a second, contralateral tubal pregnancy 11 days later.

## CASE REPORT

A 35-year-old female gravida eight para two with a recent left tubal pregnancy and a left salpingectomy 11 days prior, presented to the emergency department (ED) with two days of left lower and upper quadrant abdominal pain radiating to the epigastric region. The patient’s last menstrual period had been several months prior. She had a family history of ectopic pregnancies and a history of a prior sexually transmitted infection but no documented history of pelvic inflammatory disease. The physical examination revealed left lower quadrant abdominal tenderness, rebound, guarding, and left adnexal tenderness. Her vital signs were unremarkable, with an initial blood pressure of 132/82 millimeters of mercury, pulse of 75 beats per minute, temperature of 98°F, and oxygen saturation of 99% on room air.

**Image 1:**
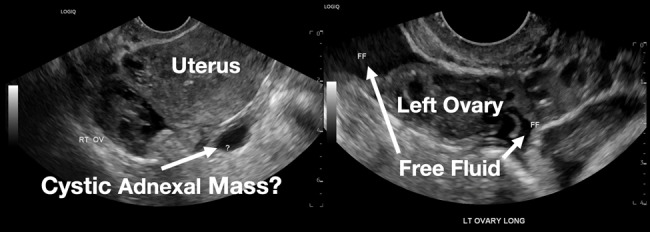
This image shows the patient’s ultrasound findings 11 days after her initial diagnosis of a left tubal pregnancy. The image on the left shows the right adnexa with an arrow pointing to a rounded cystic structure with a peripheral soft tissue component, blood flow, and free fluid. The image on the right shows the left ovary with two arrows pointing to a moderate amount of free fluid in the left adnexa.

Her laboratory studies were notable for a hemoglobin of 11.9 grams per deciliter (g/dL) (reference range: 11.6–15 g/dL) and a normal white blood cell count of 8.4 thousand per cubic milliliter (k/μL) (reference range: 5–10 k/μL). Her lipase and liver function tests were within normal limits. Her human chorionic gonadotropin (hCG) had tripled from 6,253 milli-international units per milliliter (mIU/mL) (reference range: ≤2 mIU/mL) from her prior presentation to 18,038 mIU/mL. Transvaginal ultrasound showed a possible ectopic pregnancy adjacent to the right ovary and no intrauterine pregnancy (Image 1).

*CPC-EM Capsule*

What do we already know about this clinical entity?

*Ectopic pregnancy is the most common cause of maternal mortality in the first trimester, and a bilateral tubal pregnancy is the rarest subset.*

What makes this presentation of disease reportable?

*Most reported cases of bilateral tubal pregnancies are associated with the use of assisted reproductive techniques whereas our patient had no such history.*

What is the major learning point?

*This case shows the importance of having a high index of suspicion for ectopic pregnancy in all female patients of reproductive age who present with abdominal pain.*

How might this improve emergency medicine practice?

*It serves as a reminder to keep the rare and life-threatening diagnosis of ectopic pregnancy on the differential even when the diagnosis may seem unlikely.*

**Image 2:**
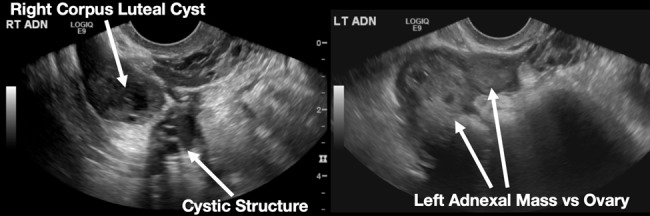
This image shows the patient’s initial ultrasound findings. The image on the right shows two arrows pointing to the left adnexa with a complex focus measuring 2.5 × 2.6 × 2.6 centimeters (cm) with a cystic focus of 0.5 cm within it and a hyperechoic thick rim. The image on the left shows the right adnexa with arrows pointing to a small, probable 0.8 cm corpus luteal cyst and an anechoic cyst measuring 1.6 cm.

**Image 3:**
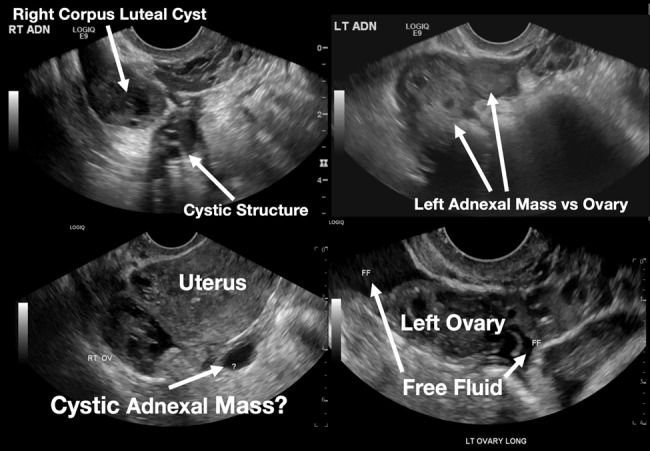
This image shows both of the patient’s ultrasound findings as described above for ease of comparison. The upper panel is from the initial encounter and the lower panel is from the patient’s encounter 11 days later.

The patient declined medical abortion and was taken to the operating room for a right salpingectomy. On her initial presentation 11 days prior, the patient’s preoperative ultrasound had shown a complex focus of the left ovary with a hyperechoic thick rim suggestive of ectopic pregnancy and probable right-sided corpus luteal and anechoic cysts (Image 2, Image 3). No evidence of right ectopic pregnancy was documented intraoperatively during the patient’s initial salpingectomy. Pathology findings from the initial left- and subsequent right-sided procedures showed immature chorionic villi, congestion, and hemorrhage consistent with a tubal pregnancy. The patient had an uneventful recovery after her second surgery.

## DISCUSSION

A preoperative diagnosis of bilateral ectopic pregnancy is neither easy nor straightforward, especially given its rarity.^5^ Ectopic pregnancy can masquerade as other conditions such as gastrointestinal disease (e.g., appendicitis), urinary tract disease, or other gynecological disorders such as ruptured cyst or ovarian torsion).^6^ This case highlights the challenges of identifying a bilateral tubal pregnancy even after obtaining the appropriate diagnostic studies.

Transvaginal ultrasound and serum hCG measurement are the first steps in diagnosing an ectopic pregnancy. Ultrasonography, however, can rarely definitively diagnose an ectopic pregnancy, as most do not advance to a stage where a gestational sac with a yolk sac or embryo is present.^7^ More commonly, a mass or a mass with a hypoechoic area is visualized and should raise suspicion for an ectopic pregnancy. These findings can also be confused with other structures, such as a paratubal cyst, corpus luteum, hydrosalpinx, endometrioma, or bowel.

Serum hCG cannot be used to distinguish between an intrauterine and ectopic pregnancy but can be used to determine whether the pregnancy has advanced enough for an intrauterine gestational sac to be visible on transvaginal ultrasound. Although there is debate regarding the best serum hCG cutoff, the conservatively high value of 3,500 mIU/mL is commonly accepted.^4^ This typically occurs around five to six weeks of gestation. Absence of a possible gestational sac in the setting of an elevated serum hCG level can lead to an increased index of suspicion for ectopic pregnancy.

Serial serum hCGs can also be used to assess the progression of early pregnancy before ultrasound findings become diagnostic.^4^ Typically, the hCG values of an intrauterine pregnancy double every 48 hours. When this does not occur, there should be an increased clinical suspicion for a miscarriage or a pregnancy that has implanted outside the uterus. Using this method of serial measurements is contingent on adequate patient follow-up and, depending on the institution, can have low utility in the ED setting. The estimation of hCG has also not proven to be reliable for distinguishing a bilateral from a unilateral tubal pregnancy while ultrasonography has only rarely identified bilateral tubal pregnancies preoperatively. In fact, in a review of 16 case reports on bilateral ectopic pregnancies, the second pregnancy was identified on ultrasound in only six cases.^8^ Often the second pregnancy is mistaken for an ovarian cyst, as in our case, further hindering timely diagnosis and treatment.^5^,^9^–^11^

There are no risk factors specific to the development of bilateral tubal pregnancy. Even risk factors for developing unilateral ectopic pregnancies are present only about 50% of the time, rendering them of little use for increasing or decreasing a clinician’s suspicion for an ectopic pregnancy. Additionally, both a bilateral ectopic pregnancy and a pregnancy of dizygotic twins require more than one ovulation event to occur in close temporal proximity. This phenomenon, known as hyperovulation, is uncommon. In the absence of assisted reproductive technology, dizygotic twins account for approximately 70% of twin gestations, which themselves account for 3% of live births.^12^ This is, however, likely an underestimation of the prevalence of hyperovulation since most of a female’s ovulatory cycles do not result in fertilization, leaving these occurrences undetected (see Appendix A).

Clinical symptoms and physical examination findings are frequently vague in patients presenting with ectopic pregnancy. As many as one-third of women diagnosed with an ectopic pregnancy have no clinical signs, and 9% of women have no symptoms. Symptoms may include pelvic or abdominal pain, vaginal bleeding, and breast soreness between six to ten weeks of gestation.^6^ Additionally, no single physical examination finding or maneuver is specific for ectopic pregnancy although common physical examination findings include cervical motion tenderness in approximately 67% of cases, abdominal or pelvic tenderness in approximately 75% of cases, and a palpable adnexal mass in approximately 50% of cases.^6^ In instances of rupture, rebound tenderness and guarding may be appreciated on abdominal examination.

Clinical symptoms and physical examination also cannot be relied upon for distinguishing between a unilateral or bilateral ectopic pregnancy due to the innervation of the abdomen (see Appendix B). In the case of our patient, her inconsistent physical examination, which suggested left-sided pathology when her ectopic pregnancy was actually on the right, may be due to a combination of factors, including her postoperative pain being more pronounced than the discomfort caused by her ectopic pregnancy, poor localization due to abdominal innervation, or an error in physical examination documentation.

Due to the rarity of bilateral ectopic pregnancies, there is no well-established recommendation or standard of care for management. Many reported cases of bilateral ectopic pregnancies are treated with surgical intervention. It is important to note, however, that the second pregnancy is often diagnosed intraoperatively. It is theoretically possible, therefore, that some cases of presumed unilateral ectopic pregnancies that are medically treated may have been cases of bilateral tubal or heterotopic pregnancies.

Conversely, unilateral ectopic pregnancies have well-established treatment guidelines. Treatment is dependent on the patient’s hemodynamic stability, desire for future pregnancy, and patient-informed choice based on the risks and benefits of each approach. Stable patients are candidates for medical management with methotrexate, a folate antagonist that interrupts the synthesis of the purine nucleotides serine and methionine, which effectively inhibits deoxyribonucleic acid (DNA) synthesis, DNA repair, and cell replication. Contraindications to methotrexate include immunodeficiency, anemia, leukopenia, thrombocytopenia, active pulmonary disease, active peptic ulcer disease, hepatic dysfunction, renal dysfunction, breastfeeding, and inability to follow up for surveillance.^4^ Three published methotrexate protocols – single- dose, two-dose, and multiple-dose – are used to manage ectopic pregnancy. There is no consensus on which protocol is best; however, there is a well-established trend of increased effectiveness as well as a greater number of adverse effects with an increase in the number of doses. The overall treatment success of methotrexate is believed to be in the range of 70 to 90%.^4^

The surgical management of ectopic pregnancy consists of either a salpingostomy, where the contents of the fallopian tube are removed, or salpingectomy, where the fallopian tube itself is removed. Surgery is warranted when an individual has contraindications to medical management, is hemodynamically unstable, has failed medical management, or if surgical management is preferred by the patient after a discussion of the risks and benefits of treatment options.^4^ Randomized control trials have shown no statistically significant difference in the rates of subsequent intrauterine pregnancy or repeat ectopic pregnancy between the two procedures; however, cohort studies have indicated that salpingostomy is associated with a higher rate of subsequent intrauterine pregnancy and repeat ectopic pregnancy compared to salpingectomy.^13^ The decision to perform a salpingostomy or salpingectomy depends heavily on the patient’s desire for future pregnancy, clinical stability, and the extent of fallopian tube damage.

## CONCLUSION

Given the high morbidity and mortality associated with missed ectopic pregnancy, it is important to maintain a high index of suspicion in biologically female patients of reproductive age with abdominal or pelvic pain, even in the weeks following recent treatment of ectopic pregnancy. As is evidenced by this case presentation and a brief review of the literature, a diagnosis of unilateral ectopic pregnancy does not preclude an eventual bilateral diagnosis. In the ED specifically, improvement of ultrasound sensitivity and accurate interpretation may allow earlier identification and, hence, treatment of bilateral ectopic pregnancies, which may lead to a more favorable prognosis.

## Supplementary Information
