# Supplementary material for: Bilateral Tubal Pregnancies Presenting 11 Days Apart: A Case Report
Source: Clin Pract Cases Emerg Med. 2023 Feb 9;7(1):11–5. doi: 10.5811/cpcem.2022.10.56910 (PMC9983339; doi:10.5811/cpcem.2022.10.56910)
Supplement: Supplementary file 1 [file cpcem-07-011-s001.pdf]

Appendix A

Hyperovulation describes the uncommon instance where more than one follicle matures and is released by the ovary. Recent studies suggest that the ovulation of more than one egg may occur more commonly than previously thought, especially in older women and in those with a genetic predisposition. It is difficult to say with certainty what the prevalence of this phenomenon is because many of these instances are clinically asymptomatic. A 2006 study looking at follicular growth in three consecutive, spontaneous ovulatory cycles of 507 women found that 21% of the women developed two or more follicles, and by implication multiple rather than single ovulations, in more than one cycle.[1] Hyperovulation is associated with increased levels of follicle-stimulating hormone (FSH). This is seen in women who artificially increase their FSH levels during in vitro fertilization with medications such as clomiphene citrate, those with a genetic predisposition, or in older women due to lower levels of inhibin secreted by the ovaries, which has a negative feedback effect on FSH secretion from the anterior pituitary.[1] Research looking at the genetics associated with dizygotic twins, which has been determined to be a heritable trait, has found that variations in several genes increase the chances of spontaneous twinning. One such gene is Follicle Stimulating Hormone Subunit Beta (FSHB), which is involved in the release of FSH, and another is the SMAD3 gene, which regulates the response of the ovaries to FSH.[2] This suggests that the presence of two separate ectopic pregnancies, as seen in this case, may also be increased in those with these genetic variations.

Appendix B

The location of the initial perceived discomfort in patients with abdominal pathology is often vague and of poor clinical utility due to the autonomic nerve supply to the visceral

peritoneum and the somatic innervation of the parietal peritoneum. The pain sensed by the visceral peritoneum if often dull and poorly localized and is generally a response to the distention or stretching of the abdomen. Parietal peritoneum involvement, on the other hand, which receives somatic innervation from the spinal nerves, produces a localized, sharp type of pain.[3] This is classically described in cases of appendicitis where the irritation of the visceral peritoneum leads to a generalized abdominal pain that only later localizes to the right lower quadrant when the inflammation has progressed to the parietal peritoneum.

REFERENCES

1. Beemsterboer SN, Homburg R, Gorter NA, et al. The paradox of declining fertility but increasing twinning rates with advancing maternal age. *Hum Reprod*. 2006;21(6):1531-2.

2. Mbarek H, van de Weijer MP, van der Zee MD, et al. Biological insights into multiple birth: genetic findings from UK Biobank. *Eur J Hum Genet*. 2019;27(6):970-979.

3. Kalra A, Wehrle CJ, Tuma F. Anatomy, Abdomen and Pelvis, Peritoneum. *StatPearls.* https://www.ncbi.nlm.nih.gov/books/NBK534788/. Accessed July 1, 2021
